# Driving Interface Based on Tactile Sensors for Electric Wheelchairs or Trolleys

**DOI:** 10.3390/s140202644

**Published:** 2014-02-10

**Authors:** Andrés Trujillo-León, Fernando Vidal-Verdú

**Affiliations:** 1 Department of Electronics, University of Málaga, Málaga 29071, Spain; 2 Department of Electronics, University of Málaga, Biomedical Research Institute of Málaga, Málaga 29071, Spain; E-Mail: fvidal@uma.es

**Keywords:** tactile sensors, assistive technologies, power wheelchair, electric trolley

## Abstract

This paper introduces a novel device based on a tactile interface to replace the attendant joystick in electric wheelchairs. It can also be used in other vehicles such as shopping trolleys. Its use allows intuitive driving that requires little or no training, so its usability is high. This is achieved by a tactile sensor located on the handlebar of the chair or trolley and the processing of the information provided by it. When the user interacts with the handle of the chair or trolley, he or she exerts a pressure pattern that depends on the intention to accelerate, brake or turn to the left or right. The electronics within the device then perform the signal conditioning and processing of the information received, identifying the intention of the user on the basis of this pattern using an algorithm, and translating it into control signals for the control module of the wheelchair. These signals are equivalent to those provided by a joystick. This proposal aims to help disabled people and their attendees and prolong the personal autonomy in a context of aging populations.

## 1. Introduction

The population in western developed countries is aging quickly. This has consequences in daily and working life [1]. It is necessary that the design of devices allows the extension of the autonomy of elder and/or impaired people. This is advantageous and also has benefits in terms of self-esteem and quality of life in general.

A straightforward application of the proposed device is its use as an alternative to the attendant joystick used with electric wheelchairs. Electric wheelchairs have two motors that power the two main wheels independently to allow turning maneuvers, including sharp turns, so they are usually driven with a hand-operated joystick. However, there are cases in which the use of a joystick can be awkward or even impossible, for example, for people who have upper spinal cord injuries, those with certain diseases of the nervous system, or who are mentally disabled or visually impaired. The very old and/or those who suffer from Alzheimer's can also be considered in this group. Some studies show that 40% of patients who use a power wheelchair think it is difficult to drive, and between 5% and 9% require the assistance of another person [2]. Sometimes it is possible to improve the autonomy with smart wheelchairs that are equipped with sensors and exploit control algorithms from the field of mobile robotics [3]. This is not possible in other cases, so an attendant to drive the chair is required. In these cases, the chairs usually have so-called “joysticks” which are incorporated in the rear handle of the chair [4].

However, as already mentioned, these human-machine interfaces are not always the most appropriate tool. Some authors propose the use of a tactile screen instead [5,6]. The user touches the screen, similar to that of common smart devices nowadays, to lead the chair or indicate the destination. However, this solution often suffers from the disadvantage, described above, of the lack of easy handling and thus the incorrect interaction between man and machine, which is detrimental to the usability of the system [7].

Therefore, there is a need for an intuitive and highly usable interface for power wheelchairs. This interface can also be used to drive electric trolleys, for instance shopping trolleys [8], which will also increase and extend the autonomy of the elderly or impaired. They will even be helpful for healthy people that have to drive trolleys with heavy loads [9]. In [8,9] it is proposed that the user takes a handle or grip with his or her hands and drives the vehicle as if he or she were pushing it. However, they do not really use a direct interface between man and machine, moreover the force actually exerted on the handle is transferred to other points where force and/or torque sensors are placed. Such action can lead to problems arising from the deformation of the elastic bands or the means used to transmit the user commands. Furthermore, the deformation of these areas can have a complex behavior over time as a result of a complex dynamics of the set, and the readings of the sensors placed in the bands or elastic joints should be registered, which makes the control (from the readings of the sensors) complex. Another interesting work devoted to exploring the role of a tactile sensor as a human-machine interface is presented in [10], where manipulative forces are predicted from the information provided by the tactile images of the forearm.

The proposed device is based on a tactile sensor [11] to achieve a direct interface with the attendant. The tactile sensor provides information not only about the location of the contact points, but also about the contact force at these points, so its output is a force map or tactile image. The processing of this map gives the information required to drive the chair or trolley in an intuitive way. This paper describes two prototypes developed and tested by the authors that implement a human-machine driving interface, based on tactile sensors and their integration into an electric wheelchair. Hardware as well as algorithmic issues and results that show the feasibility of the proposal are presented.

## 2. Hardware of the Tactile Based Human-Machine Driving Interface

**Figure 1.**
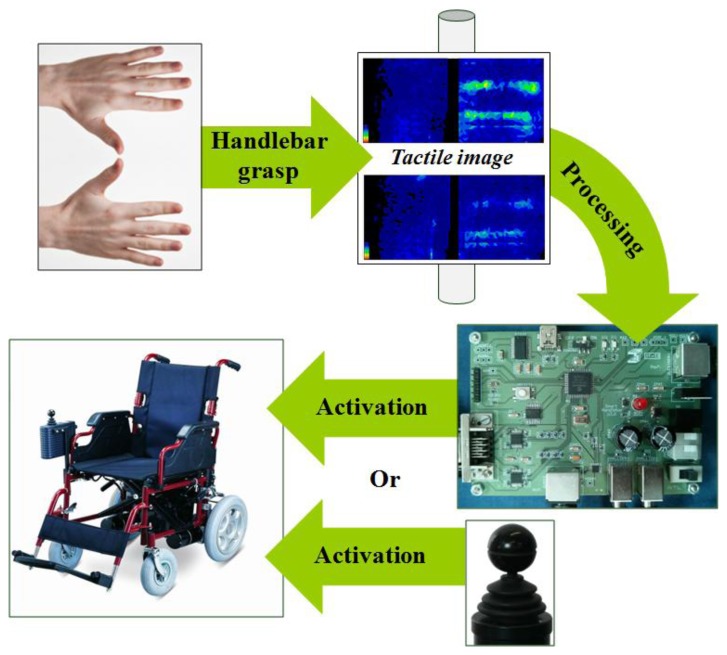
System scheme.

The basic operation of the developed system is illustrated in Figure 1. The person who drives the wheelchair or trolley grasps the handlebar and the resulting force map is registered by the tactile sensor. This information is processed by a microcontroller and different patterns are extracted from the force map. These patterns are associated with user intention. For instance: accelerate, decelerate, turn to the left, *etc*. According to the intention detected (and other parameters which will be explained in Section 3), the control electronics generates the appropriate signals in order to activate the wheel motors and make the chair move. The system provides output signals similar to those generated by the joystick which is incorporated by power wheelchairs.

### 2.1. Tactile Sensor

The tactile sensor can be a discrete array of force sensors or can be made as a single sensor matrix using many different technologies (piezoresistive, capacitive, optical, *etc*.) [12]. A common and low cost realization consists of an array of electrodes with a conductive rubber or polymer placed atop [13].

**Figure 2.**
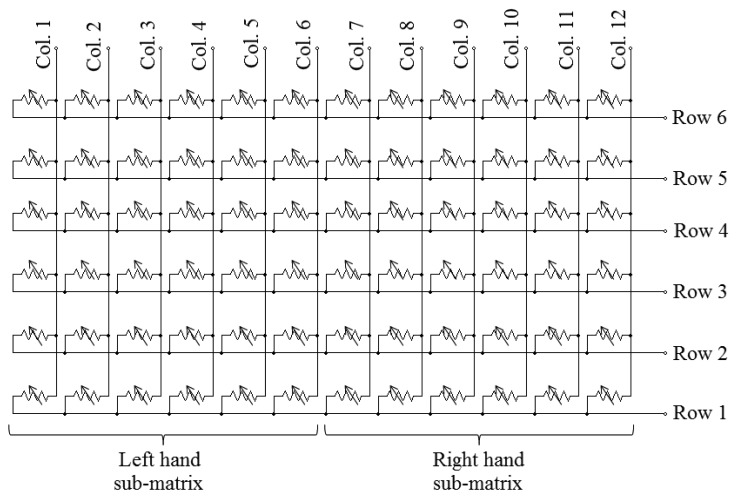
Piezoresistive matrix of the first prototype of tactile sensor.

In the case of the first prototype of this paper, an array of piezoresistive sensors has been used (see Figure 2). Its size is 6 × 12 elements (two sub-arrays of 6 × 6 elements, one per hand). Each *tactile element* (tactel) works as a variable resistor so that the higher the force applied, the lower its electrical resistance. The signal conditioning electronics scans the array and provides a force value for every tactel, so the force map can be built.

**Figure 3.**
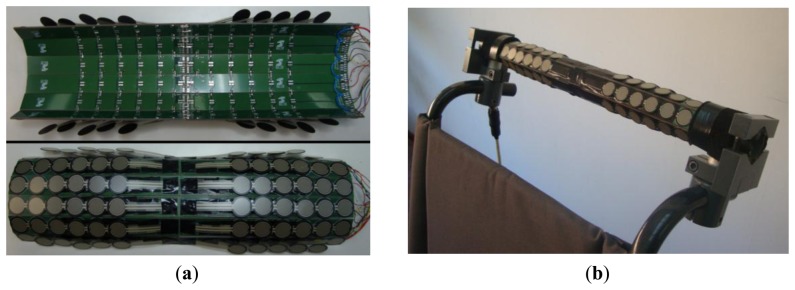
First prototype of the proposed device. (**a**) Raw tactile sensor prior to embracing the handlebar; (**b**) resulting implementation.

Figure 3 shows the implementation of the matrix of Figure 2. It is composed of one rigid Printed Circuit Board (PCB) per row in each sub-array. Then the PCBs are joined together with soldered flexible tinned bridges that make the columns of the matrix (see Figure 3a). The tactels are commercial FSR 402 force sensors [14] from Interlinks Electronics (Camarillo, CA, USA), soldered on the upper side of the PCBs. They must lie on a flat surface as they are sensitive to folds that cause undesired interferences [10]. The assembled structure is mounted, embracing the wheelchair handlebar, as can be seen in Figure 3b.

**Figure 4.**
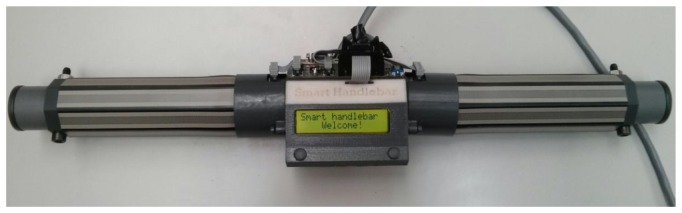
Second prototype of the proposed device.

This first prototype was used to carry out several experiments that focused on knowing how the force maps evolve while the chair is being driven by an attendant. However, the tinned bridges that join the PCBs are fragile and the structure had to be frequently taken apart for repair. Therefore, a second prototype was developed to overcome these drawbacks and incorporate some improvements. The array of this second prototype is simpler as it is composed of two sub-arrays, one for each hand, of only eight elements each (see Figure 4). The force sensors are now of longitudinal shape (Interlink Electronics FSR 408 [15]) and are placed on the flat faces of an octagonal bar. It is sturdier as there is no soldering under the pressure sensitive area. Therefore, lifetime related to wear and tear, due to physical contact, must be similar to that of the force sensor, which is 10 million actuations. Moreover, since it has fewer force sensors this implementation is cheaper and has a quicker response time than the first one. An LCD was also added to show messages to the user.

### 2.2. Control Electronics

**Figure 5.**
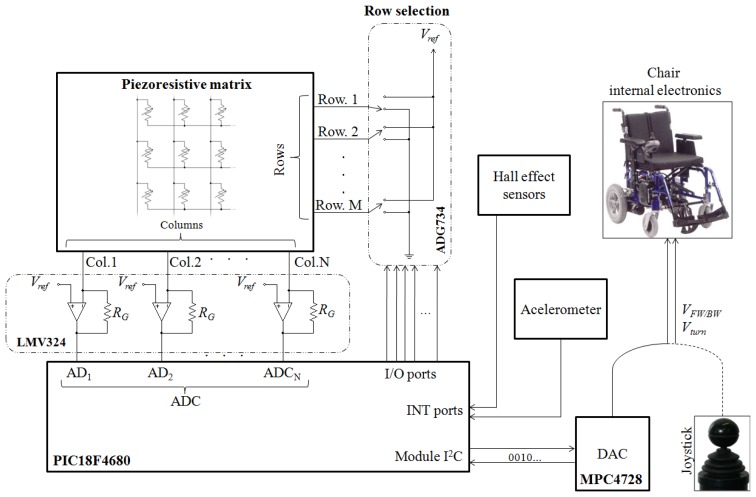
Control electronics scheme.

Figure 5 shows the schematic of the control electronics. The rows of the matrix are connected to analog switches (ADG734, Analog Devices, Norwood, MA, USA) and the columns to transimpedance amplifiers (based on LMV324 operational amplifiers, Texas Instruments, Dallas, TX, USA). A microcontroller (PIC18F4680) scans the array by closing the switches sequentially through general purpose I/O ports. The addressed row is grounded while the other rows remain connected to a reference voltage (*V_ref_*). This is also the voltage at the non-inverting input of the amplifiers. In this way parasitic resistive paths are short circuited and crosstalk is canceled. The output voltage of the transimpedance amplifiers provides the force exerted on the tactels of the selected row. This voltage is digitalized by the analog-to-digital converter of the microcontroller and the force map is stored.

The force map is processed by the microcontroller as described in the following section and two outputs are generated. They are converted into two analog voltage signals, *V_turn_* and *V_FW/BW_*, by a digital-to-analog converter (MPC4728, Microchip Technology Inc., Chandler, AZ, USA). As mentioned at the beginning of this section, the device works as a substitute for a commercial joystick and their outputs are similar, so they are valid inputs for the wheelchair electronics that carries out the steering. Although the prototype generates analog output signals, a direct interface with a digital bus could also be developed.

**Figure 6.**
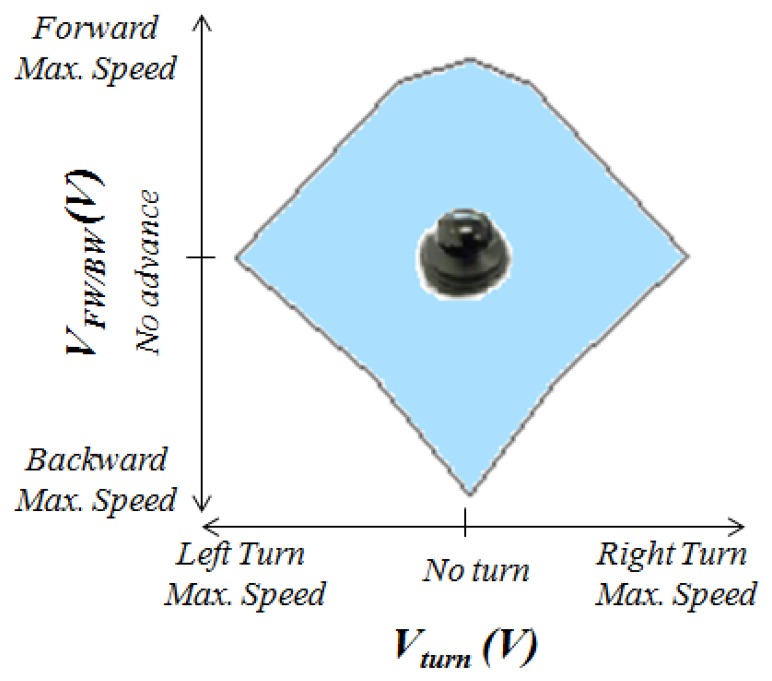
Range and meaning of joystick output voltages.

Figure 6 illustrates the relationship between the output voltages *V_turn_* and *V_FW/BW_* and the movement of the chair. The voltage *V_turn_* is proportional to the turn speed and *V_FW/BW_* is proportional to the speed in the forward/backward direction. Therefore, the larger the output voltages the higher the speed. A forward displacement is achieved by increasing the voltage *V_FW/BW_* while keeping the voltage *V_turn_*. On the other hand, a backward movement is achieved if the voltage *V_FW/BW_* is decreased while the value of *V_turn_* does not change. In addition, a value of *V_turn_* above or below zero will cause a higher speed of the left wheel than that of the right wheel or *vice versa*, so a turn is carried out. Finally, the output voltages of a joystick and also of the proposed device are limited inside a certain region (bluish area in Figure 6) to guarantee safe driving.

**Figure 7.**
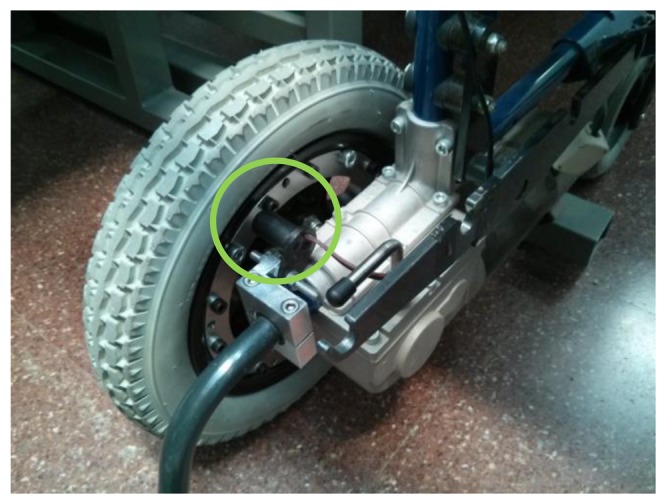
Incremental encoder based on Hall effect sensor to implement odometry.

Some auxiliary elements have been added to the system to help in the testing procedure. Specifically, incremental encoders have been implemented with Hall effect sensors and rings of bolts in both wheels (see Figure 7) to obtain information about the trajectory, speed, *etc*. An accelerometer has also been added to detect collisions and register vibrations.

**Figure 8.**
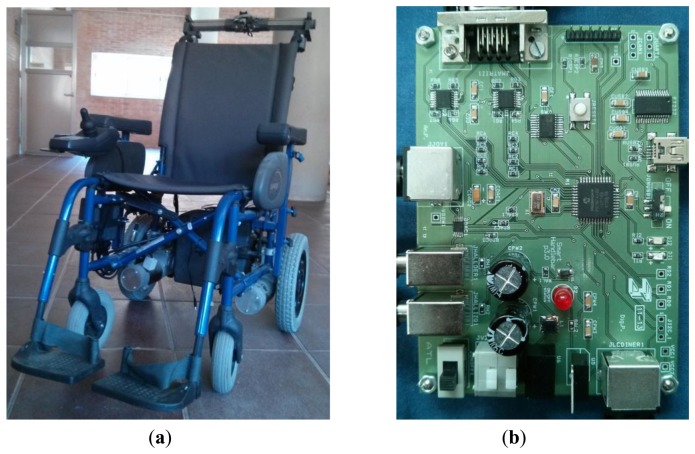
(**a**) Prototype mounted in a power wheelchair; (**b**) Control electronics.

Figure 8a shows a picture of the developed prototype and Figure 8b shows a photo of the control electronics based on a microcontroller. The electronics has not been optimized regarding the input-output delay nor power consumption, because the main purpose of this work is to demonstrate the feasibility of the approach. The power supply for the device is taken from the batteries of the wheelchair. Power consumption of the device of the second prototype is around 40 mA. Taking into account that electric motors consume as much as 45 A each, this is not a main issue in the design. Nevertheless, further improvements in this aspect can be made by choosing low power integrated circuits and even tactile sensors based on a different technology.

## 3. Generation of the Haptic-Based Movement

**Figure 9.**
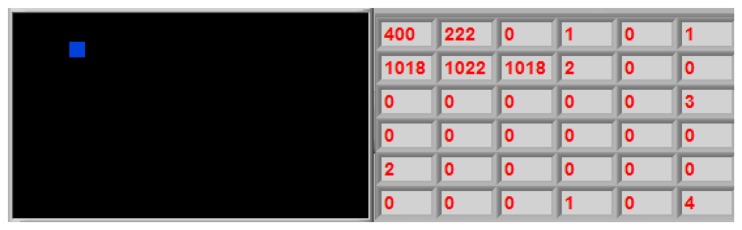
Center of mass of a tactile image.

A common processing of the tactile image or map of forces exerted by the user is the calculation of its center of mass. The center of mass of the tactile image provided by a sensor with *N* × *M* sensing units is defined as:

(1) $$\begin{array}{ll} C_{x} & =\frac{\sum_{x=1}^{M}\sum_{y=1}^{N}x\cdot f(x,y)}{\sum_{x=1}^{M}\sum_{y=1}^{N}f(x,y)}\\ C_{y} & =\frac{\sum_{x=1}^{M}\sum_{y=1}^{N}y\cdot f(x,y)}{\sum_{x=1}^{M}\sum_{y=1}^{N}f(x,y)} \end{array}$$

where *x* is the row number, *y* the column number, *f*(*x*,*y*) the force value at the coordinates indicated by *x* and *y*, and the point (*Cx*,*Cy*) is the center of mass. By way of example, Figure 9 shows the resulting array from the reading of a tactile sensor and its center of mass computed.

**Figure 10.**
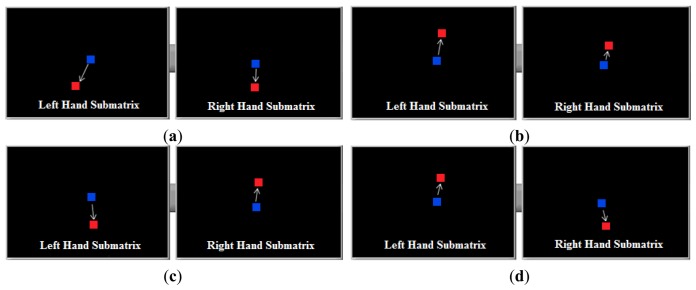
Displacement of the center of mass under different maneuvers registered with the prototype in Figure 3: (**a**) forward movement; (**b**) backward movement; (**c**) turn to the right; and (**d**) turn to the left.

It was observed that the center of mass of the tactile images obtained by two tactile sensors placed on the left and right side of the handle moves when the user tries to make a maneuver, and that these shifts can be interpreted. For example, Figure 10a–d shows the displacement of the center of mass (wherein the tactile sensor is divided into two, one for the left and the other for the right hand), when they perform a movement of the vehicle: forward, backward, right turn and left turn respectively. These results were obtained with the first prototype of the device, described in Section 2.1.

Although all the information provided by the tactile image can be used, for instance the size of the hand to adapt the driving, only the coordinates of the center of mass are used in these first prototypes. Actually, Figure 10 shows that the different maneuvers can be identified from only the shifts of the *y* coordinate. This has been exploited in the development of the second prototype described in Section 2.1, where both sub-matrices were built as linear arrays and the spatial resolution along the *y* axis was increased. Below, the algorithm to obtain the proper *V_turn_* and *V_FW/BW_* voltages from the shifts of the center of mass in these sensors is described.

**Figure 11.**
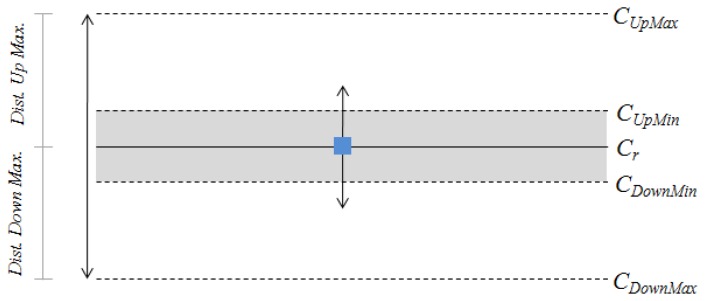
Parameters of the input space used by the algorithm.

When the user grasps the handlebar, the centers of mass are computed and stored. These will be considered the centers of mass in rest condition, *i.e.*, the user is only grasping the handlebar with no intention of moving the wheelchair or trolley. These points, *C_Lr_* for the left hand and *C_Rr_* for the right hand, will be used as references to determine if the centers of mass of the subsequent readings are shifted or not. Moreover, a dead band around *C_Lr_* and *C_Rr_* is defined (shaded zone in Figure 11). As long as the current center of mass, *C_L_* or *C_R_*, is inside this area it will be considered to be in rest condition. This prevents little displacements around these references, for instance those due to electrical noise or mechanical vibrations, which may make the algorithm work improperly.

In Figure 11, *C_UpMin_* and *C_DownMin_* represent the upper and the lower limits of the dead band, respectively. *C_UpMax_* and *C_DownMax_* refer to the limits of the center of mass excursion during the driving and are found heuristically in the prototypes of this paper.

**Figure 12.**
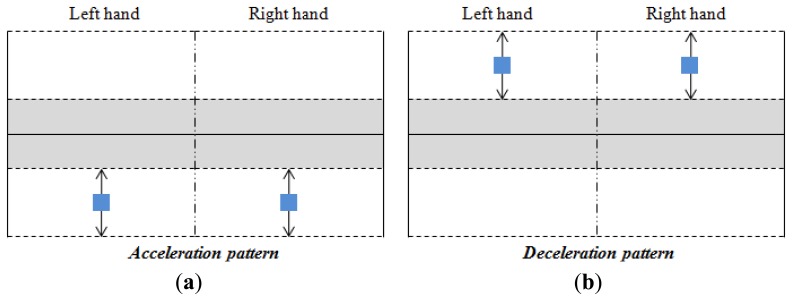
(**a**) Acceleration and (**b**) deceleration patterns.

**Figure 13.**
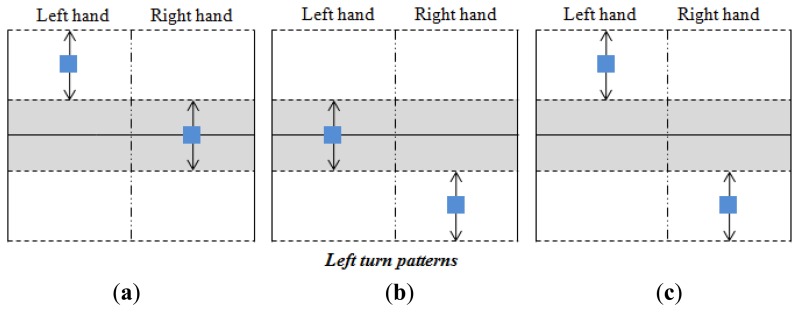
Left turn patterns.

**Figure 14.**
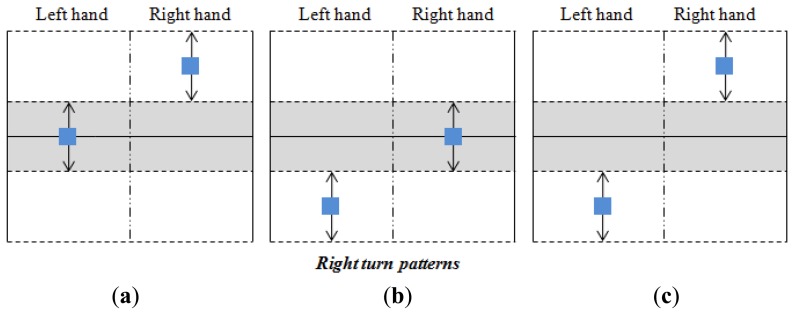
Right turn patterns.

Taking into account the shifts shown in Figure 10 and the parameters described above, the classification of the user's intention is carried out as Figures 12,13 and 14 illustrate. The blue squares represent the centers of mass and the arrows indicate the range of possible displacement in which the intention of the user is considered. Note that the information of the centers of mass of both hands is essential to identify the purpose of the user.

Note also that there is only one pattern identified with acceleration or deceleration (see Figure 12), while there are three patterns related to an intention to turn (see Figures 13 and 14). The reason is that the user can make a turn by changing the force exerted by both hands, or only that exerted by one hand. However, both hands are always used when an increment or decrement of the advance speed is desired. Note that each pattern is associated to only one purpose of the user.

### 3.1. Acceleration/Deceleration Processing

Acceleration and deceleration processing is carried out when the patterns of Figure 12 are detected. If the acceleration (see Figure 12a) or the deceleration (see Figure 12b) patterns are detected, the speed of advance of the wheelchair is calculated as:

(2) $$S_{\text{ad}v_{t}}(C_{L},C_{R})=\{\begin{array}{l} S_{\text{ad}v_{t-1}}+\Delta_{\max}\cdot \Delta_{\text{acc}}(C_{L},C_{R}),\text{if an acceleration pattern is detected}\\ S_{\text{ad}v_{t-1}}-\Delta_{\max}\cdot \Delta_{\text{dec}}(C_{L},C_{R}),\text{if a deceleration pattern is detected} \end{array}$$

where *S*_*adv*_*t−1*__ represents the current forward speed of the wheelchair, Δ_max_ is the maximum increment of the speed allowed, Δ*_acc_* is given by:

(3) $$\Delta_{\text{acc}}(C_{L},C_{R})=\frac{\left(C_{L_{\text{DownMin}}}+C_{R_{\text{DownMin}}})-(C_{L}+C_{R}\right)}{\left(C_{L_{\text{DownMin}}}+C_{R_{\text{DownMin}}})-(C_{L_{\text{DownMax}}}+C_{R_{\text{DownMax}}}\right)}$$

and Δ*_dec_* is obtained from the expression:

(4) $$\Delta_{\text{dec}}(C_{L},C_{R})=\frac{\left(C_{L}+C_{R})-(C_{L_{\text{UpMin}}}+C_{R_{\text{UpMin}}}\right)}{\left(C_{L_{\text{UpMax}}}+C_{R_{\text{UpMax}}})-(C_{L_{\text{UpMix}}}+C_{R_{\text{UpMin}}}\right)}$$

Note that the change rate of *S_adv_t__* is determined by Δ*_acc_* and Δ*_dec_*. These parameters are inside the interval (0, 1] and depend on how far the centers of mass, *C_L_* and *C_R_*, are from the dead band. Thus, the largest increment of the current speed takes place when *C_L_* reaches the value C*_L_DownMax__* and *C_R_* the value *C_R_DownMax__*, while the minimum increment happens when *C_L_DownMin__* is *C_L_* and *C_R_* is *C_R_DownMin__*. The behavior is similar when the speed is decremented but now the change rate depends on the parameters *C_L_UpMax__*, *C_R_UpMax__*, *C_L_UpMin__* and *C_R_UpMin__* as the centers of mass shift toward their upper limits in this scenario.

Finally, the voltage *V_FW/BW_* in Figure 6 is given by:

(5) $$V_{FW/BW}(C_{L},C_{R})=A\cdot S_{\text{ad}v_{t}}(C_{L},C_{R})+B$$

where *A* and *B* are constants required to adapt the output range of *V_FW/BW_* to the input range of the wheelchair electronics that controls the motors.

It is worth highlighting that the user does not have to exert any force in order to maintain a fixed speed (in this case *S_adv_t__* = *S*_*adv*_*t−1*__). It is thought that this is the most comfortable approach since the scenario in which the chair moves forward at a steady speed is the most frequent.

### 3.2. Turn Processing

A turn is detected if one of the patterns of Figures 13 and 14 is registered. In this case, the turn speed of the turn to the left is obtained by:

(6) $$S_{L}=\Delta_{T_{\max}}\cdot \Delta_{L}(C_{L},C_{R})$$

where Δ_*T*_max__ is the maximum turn speed and:

(7) $$\Delta_{L}={\left(\frac{\left(C_{L}‐C_{R})‐(C_{L_{\text{UpMin}}}‐C_{R_{\text{DownMin}}}\right)}{\left(C_{L_{\text{UpMax}}}‐C_{R_{\text{DownMax}}})‐(C_{L_{\text{UpMin}}}‐C_{R_{\text{DownMin}}}\right)}\right)}^{2}$$

Similarly, the turn speed to the right is computed as:

(8) $$S_{R}=\Delta_{T_{\max}}\cdot \Delta_{R}(C_{L},C_{R})$$

where:

(9) $$\Delta_{R}={\left(\frac{(C_{R}‐C_{L})‐\left(C_{R_{\text{UpMin}}}‐C_{L_{\text{DownMin}}}\right)}{\left(C_{R_{\text{UpMax}}}‐C_{L_{\text{DownMax}}}\right)‐\left(C_{R_{\text{UpMin}}}‐C_{L_{\text{DownMin}}}\right)}\right)}^{2}$$

**Figure 15.**
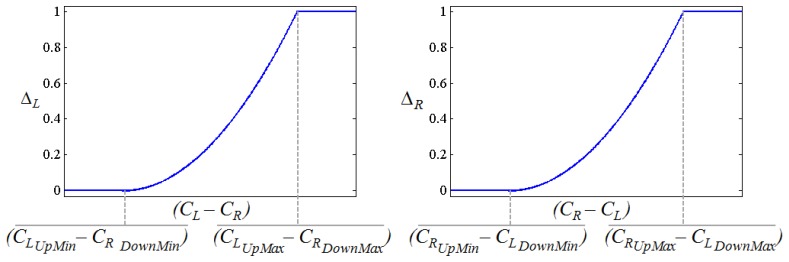
Δ*_L_* and Δ*_R_* functions.

Δ*_L_* and Δ*_R_* are inside the interval (0, 1] and they determine how tight the turn is. They are depicted as Figure 15 where their dependence on the Euclidean distance between *C_L_* and *C_R_* can be seen. This dependence follows a quadratic behavior to make the turns smoother (low pass filtering is also performed with the same goal). Note that the larger the distance ∣*C_R_* - *C_L_*∣ the tighter the turn will be. Note also that ∣*C_R_* - *C_L_*∣ is smaller in the patterns of Figures 13a,b and 14a,b while the tightest turn is achieved with the patterns in Figures 13c and 14c.

Finally, the voltage *V_turn_* in Figure 6 is given by the expression:

(10) $$V_{\text{turn}}(C_{L},C_{R})=\{\begin{array}{l} C\cdot S_{L}(C_{L},C_{R})+D,\text{if a turn to the left is detected}\\ C\cdot S_{R}(C_{L},C_{R})+D,\text{if a turn to the right is detected} \end{array}$$

where *C* and *D* are constants required to adapt the output range of *V_turn_* to the input range of the wheelchair electronics that controls the motors.

### 3.3. Advance Speed Correction

**Figure 16.**
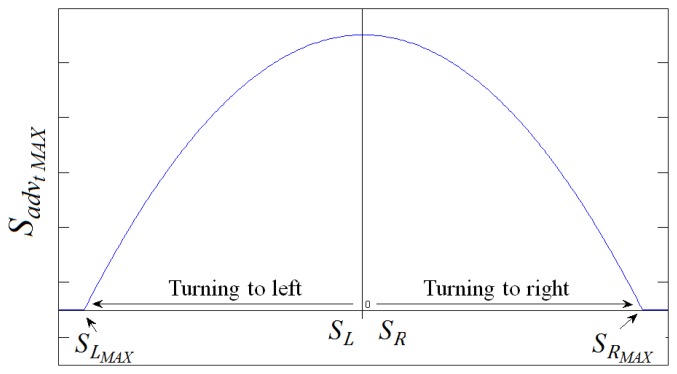
Advance speed correction according to the turn speed.

Once the turn speed is calculated, the advance speed has to be modulated by its value. This prevents the dangerous scenario where a tight turn is made while the chair is moving forward at a high speed. Figure 16 shows how the advance speed is rated to a value that depends on the turn speed. A sort of “safe operating” area is defined below the curve in Figure 16. The final value of the advance speed is obtained from Equation (2) if this value is inside this area for a given turn speed. On the contrary, if this value is outside the safe operating area it is replaced by the rated value given by the curve in Figure 16.

## 4. Results and Discussion

**Figure 17.**
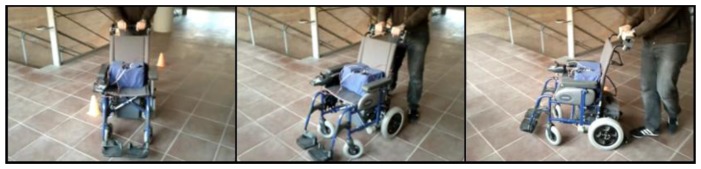
Sequence of pictures to illustrate a turn to the right.

**Figure 18.**
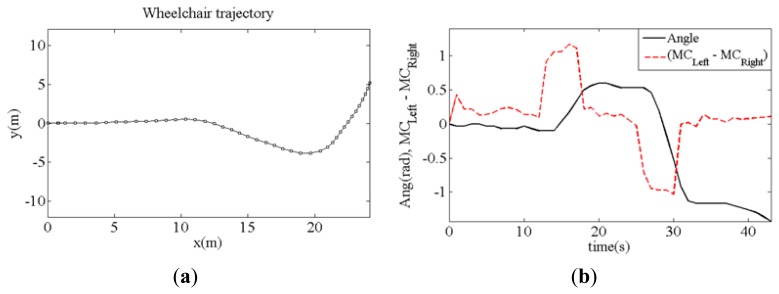
(**a**) Sample trajectory of the wheelchair obtained with odometry and evolution of the center of mass difference (left center minus right center) and (**b**) the turn angle of the chair in time.

Different tests have been carried out to demonstrate the feasibility of the proposal where the wheelchair was driven avoiding obstacles. Figure 17 shows a sample of these tests where a turn to the right is performed. Figure 18a shows the trajectory of the wheelchair as it is built with data from the Hall sensors and Figure 18b shows the displacement of the center of mass (dashed line) when the chair turns to the right and then to the left, as the trajectory depicts. The continuous line in Figure 18b shows the turning angle of the chair, as computed from the data provided by the sensors.

**Figure 19.**
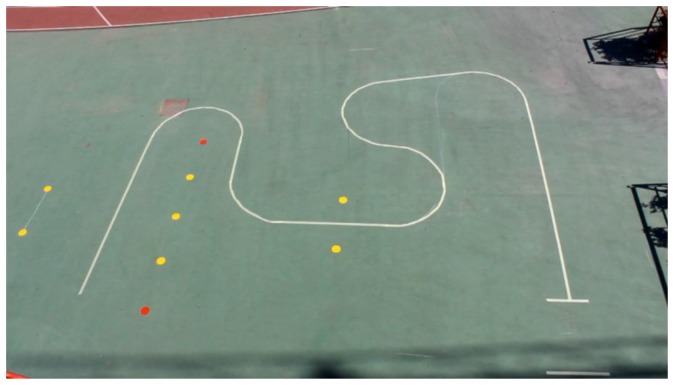
Path used to carry out the tests.

**Figure 20.**
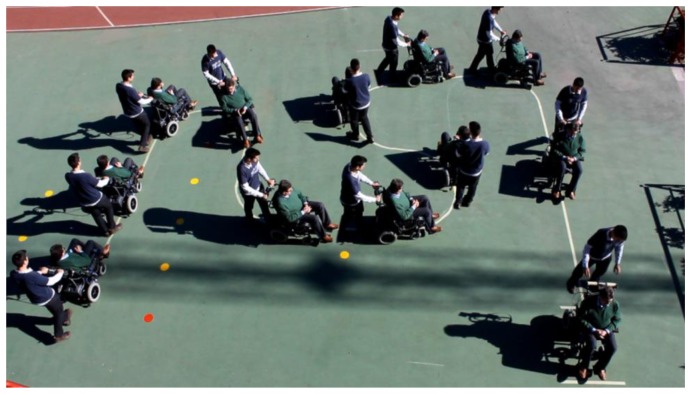
Test circuit: first user.

**Figure 21.**
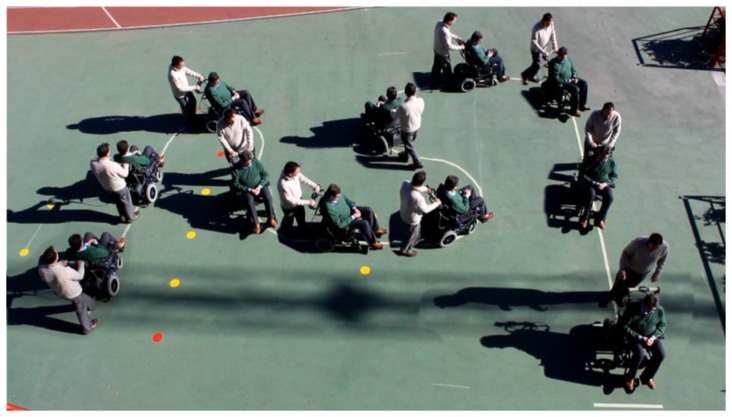
Test path: second user.

**Figure 22.**
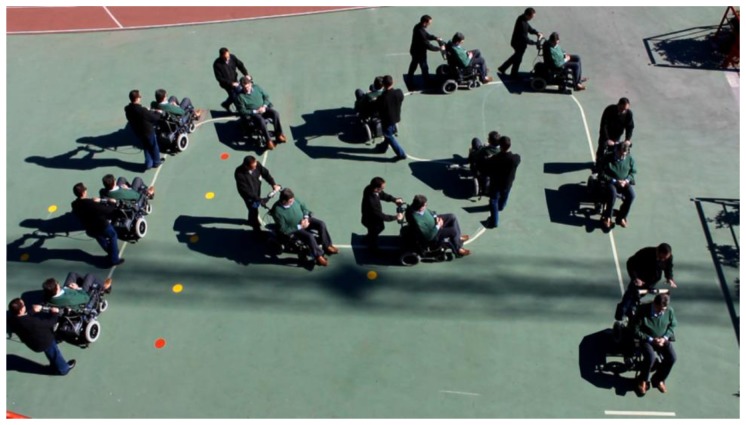
Test path: third user.

**Figure 23.**
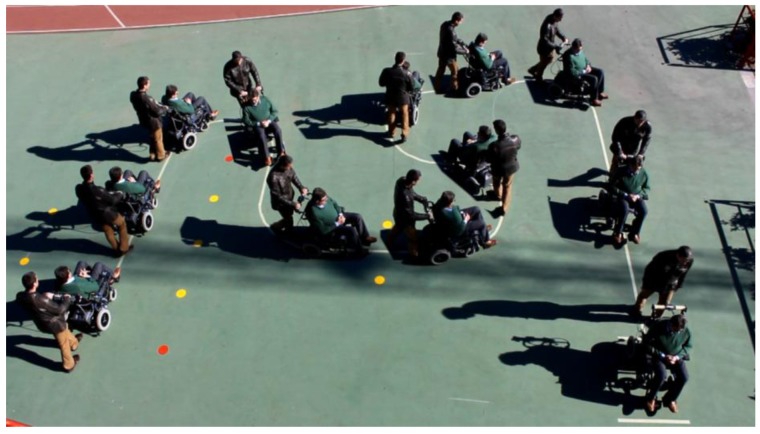
Test path: fourth user.

**Figure 24.**
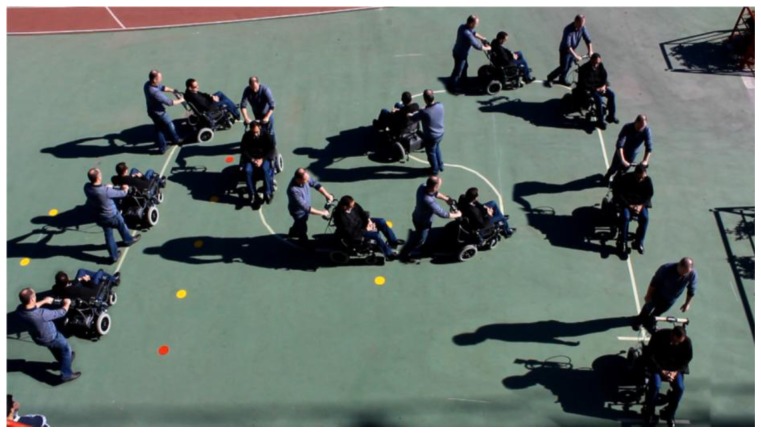
Test path: fifth user.

**Figure 25.**
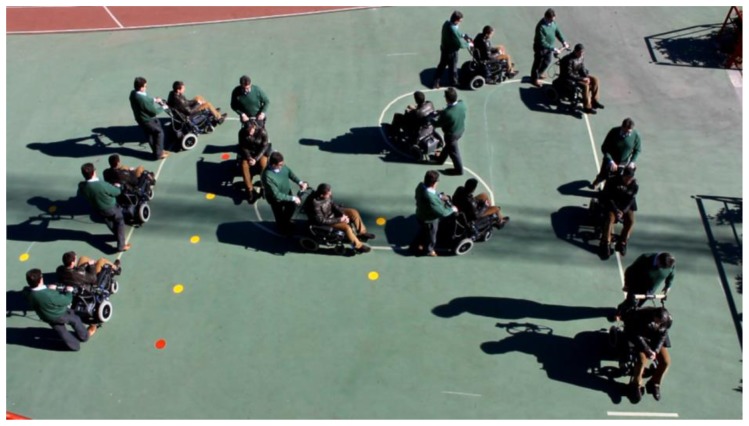
Test path: sixth user.

To see its performance under different conditions, six volunteers were asked to drive the chair following the path shown in Figure 19. Note that this path has tight turns to the left and right. The results can be seen in videos attached to this paper. Moreover, Figures 20, 21, 22, 23, 24 and 25 summarize them by superimposing a few selected frames. The resulting photograph shows the sequence of the movement along the path. In these tests, the user of Figure 20 had experience in driving the chair. The users of Figures 21 and 22 had driven the chair in different contexts a few times, for periods below 15 min in total.

**Figure 26.**
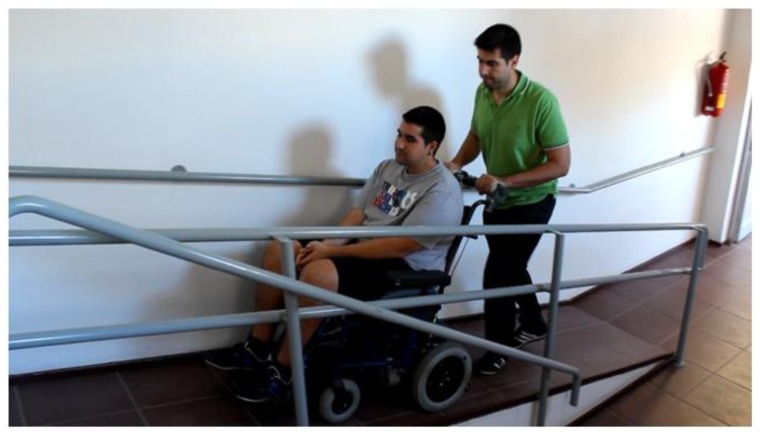
Driving on a ramp.

The users in Figures 23, 24 and 25 had no experience in driving the chair, although they were allowed to follow the path twice before the results were taken. Finally, Figure 26 shows a frame of another attached video where the wheelchair is driven on a ramp.

Generally speaking, the users found the driving quite comfortable, although some flaws were reported. The performance depends on the “quality” of grasping, which is logical taking into account the working principle of the device. If the grasping is too loose, this is interpreted as an absence of grasping and the chair stops. The initialization procedure, once the grasping is reestablished, takes a few seconds using this prototype and this is a bit confusing for the users. If the grasping is too tight, the output of the sensor is saturated and the centers of mass do not change, so driving is not possible. Sometimes there are little, sudden accelerations that can confuse the attendant, and could be uncomfortable for the user sitting in the chair. The turn speed can even occasionally be perceived as excessive by the attendant, though it is safe because of the modulation described in Section 3.3. Finally, it is assumed that the posture of the hands remains unaltered once the initial grasping is established, which could also be felt as unnatural by the driver who would maybe like to drive the chair with one hand in long forward movements. Taking these flaws into account, an improved prototype of the device is currently being developed. Information from other sensors is to be used by the algorithm that computes the speed and artificial intelligence being applied, although this work is still in progress and is currently not mature enough.

## 5. Conclusions

A device to assist in the driving of wheelchairs or trolleys has been presented. The results show the feasibility of the proposal. The driving of the vehicle is very intuitive. In fact, it is done as if it were being pushed by the attendee (instead of propelled by motors), but requiring less effort. Hence, the usability is high. The handlebar of the vehicle where the device is attached does not have to be moved nor transmit any force or torque to another point where other sensors are located, so the design and control are simpler than in other approaches. The device detects whether there is physical contact with the driver, which, from a safety point of view, can be exploited to lock the vehicle if there is no contact. Finally, the tactile sensor in Figures 3 and 4 can also be used as an input interface, for instance to set different modes of operation or parameters related to the user.

Nevertheless, the main goal is the development of a very intuitive interface that is very easy to use by elderly people and under different circumstances and environments. The performance of the device in this sense is good, but it can be improved. This is currently being done by adding more sensors and using artificial intelligence.

## Supplementary Material

One video per figure in Section 4 related to the experiments with different users is added as supplementary material. The explanation of the content of the videos is the same that this section provides for that of Figures 20, 21, 22, 23, 24, 25 and 26.
